# Perception and Signaling of Strigolactones

**DOI:** 10.3389/fpls.2016.01260

**Published:** 2016-08-23

**Authors:** Marek Marzec

**Affiliations:** ^1^Department of Genetics, Faculty of Biology and Environmental Protection, University of Silesia, KatowicePoland; ^2^Department of Physiology and Cell Biology, Leibniz Institute of Plant Genetics and Crop Plant Research, SeelandGermany

**Keywords:** strigolactone (SL), signal transduction, signal perception, GR24, SCF complex

## Abstract

Strigolactones (SLs), a recently discovered class of phytohormones, are important regulators of plant growth and development. While the biosynthetic pathway of these molecules is well documented, until recently there was not much known about the molecular mechanisms underlying SL perception and signal transduction in plants. Certain aspects of their perception and signaling, including the hormone-mediated interaction between receptor and F-box protein, degradation of suppressor proteins and activation of transcription factors, are also found in other phytohormones. However, some of SL signaling features seem to be specific for the SL signaling pathway. These include the enzymatic activity of the SL receptor and its destabilization caused by SLs. This review summarizes the current knowledge about SL signaling pathway in plants.

## Introduction

Strigolactones (SLs) are carotenoide-derived phytohormones that were originally identified as rhizosphere signal molecules, involved in parasitic and symbiotic interactions between plant roots and parasitic seeds/fungi (reviewed by Zhang et al., 2015). To date, more than 20 naturally occurring SL derivatives have been described (Al-Babili and Bouwmeester, 2015) fulfilling a plethora of roles in plant growth and development (reviewed by Obando et al., 2015). In 2008, SLs were identified as crucial regulators of plant branching (Gomez-Roldan et al., 2008; Umehara et al., 2008). In the following years it has been shown that SLs are also involved in regulating root development (Koltai and Kapulnik, 2014; Sun et al., 2016b), leaf senescence (Yamada and Umehara, 2015), and responses to nutrient stress (Marzec et al., 2013; Sun et al., 2016a), while a potential role in response to biotic stresses was recently proposed (Marzec and Muszynska, 2015).

**FIGURE 1:**
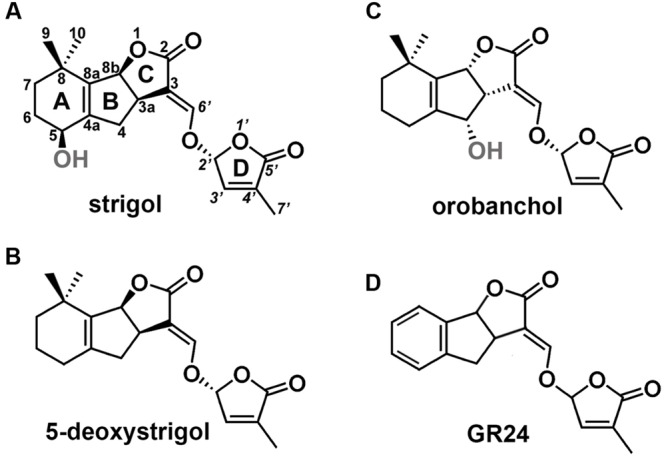
**Structures of SLs. (A)** Structure of strigol, the first identified representative of SLs, **(B)** structure of 5-deoxystrigol, the precursor of other β-oriented C-ring SLs (strigol-configured SLs), **(C)** structure of orobanchol, an example of SLs that carries an α-oriented C-ring (orobanchol-configured SLs), **(D)** structure of GR24, the synthetic analog of SLs.

Studies on mutant plants of *Arabidopsis thaliana* L., *Oryza sativa* L., *Pisum sativum* L., and *Petunia hybrida* L. enabled the identification of key proteins involved in SL biosynthesis and signaling. Biosynthesis of SL starts with the conversion of all-*trans*-β-carotene into carlactone (CL). This process takes place in plastids and involves a carotenoid isomerase and two carotenoid cleavage dioxygenases (Alder et al., 2012). Following its transport into the cytoplasm, MAX1-type monooxygenases transform CL into carlactonic acid, that is later converted into 5-deoxystrigol or orobanchol, two main precursors of other SLs (Seto et al., 2014). SLs consist of a tricyclic lactone (ABC ring) connected to a butenolide group (D ring). The C-D part is conserved among all SLs, while the A-B rings are subjected to modifications, including substitutions of the methyl, hydroxyl, and acetyloxyl groups (**Figure 1**). Based on the steric orientation of the α- (orobanchol-configured) or β-oriented (strigol-configured) C-ring SLs have been divided into two groups (Xie et al., 2013).

In contrast to the biosynthesis pathway, knowledge about the SL signaling remained limited. Recent studies, however, brought great progress in uncovering the SL signaling mechanisms and components involved in SL perception, signal conversion and downstream responses in plants.

## SL Perception

**FIGURE 2:**
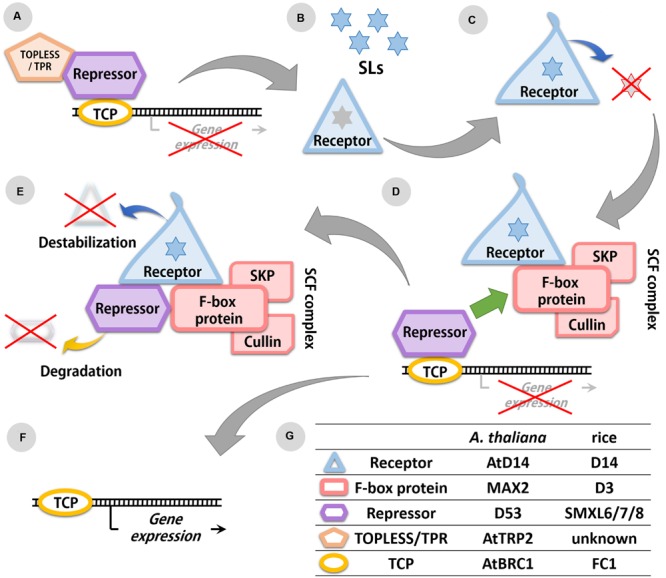
**Scheme of the SL signaling pathway. (A)** Expression of transcription factors (TFs) from the TCP family is repressed in the absence of SLs. **(B)** SL molecules are recognized by the SL receptor D14/DAD2. **(C)** The receptor hydrolyses SL molecules resulting in conformation changes of the D14/DAD2 protein. **(D)** In presence of SLs, the receptor with altered conformation is able to bind the F-box protein (MAX2/D3) from the SCF complex and the SL repressor (D53/SMXL6 to 8). **(E)** The repressor is degraded in the proteasome, also receptor is destabilized because of its changed conformation. **(F)** Degradation of repressor allows the expression of TFs from the TCP family. **(G)** List of identified components of the SLs signaling pathway in rice and *A. thaliana.*

Analysis of SL-insensitive mutants enabled the identification of potential SL receptors in various plant species: D14 in rice (Arite et al., 2009), AtD14 in *A. thaliana* (Waters et al., 2012), DAD2 in petunia (Hamiaux et al., 2012), HvD14 in *Hordeum vulgare* L. (Marzec et al., 2016), and PtD14 in *Populus trichocarpa* Torr. & A. Gray (Zheng et al., 2016). All these receptors are members of the α/β-hydrolase family and are able to bind and hydrolyze SL molecules *in vitro* (Kagiyama et al., 2013; Nakamura et al., 2013). The enzymatic activity of the D14/DAD2 protein depends on the presence of the catalytic Ser/His/Asp triad (Hamiaux et al., 2012). In DAD2, substitution of the Ser96 by Ala resulted in a loss of catalytic activity and SL perception (Hamiaux et al., 2012). X-ray crystallography analysis of the D14/DAD2 protein revealed that the Ser within the catalytic triad is also involved in binding the D ring of SLs (Zhao et al., 2013). When the SL molecule is attached to the D14/DAD2, a nucleophilic attack separates the ABC part of the SL molecule from the D ring (Scaffidi et al., 2012). This reaction also results in a change of the D14/DAD2 conformation (Nakamura et al., 2013), which is crucial for the interaction of this protein with other components of the SL signaling complex (Zhao et al., 2015) (**Figure 2**). The binding pocket of D14/DAD2 is partially covered by a cap formed by four helicases (Kagiyama et al., 2013; Nakamura et al., 2013). Studies on the barley mutant *hvd14.d* revealed that the loss of function may be also due to a reduction of the aperture of entry to the binding pocket of the D14/DAD2 protein (Marzec et al., 2016). It has to be highlighted that the D14/DAD2 protein is a specific receptor for SLs, since karrikins and other regulators of plant growth and development that are structurally similar to SLs, are not recognized by this protein (Waters et al., 2012). The dynamics by which the D14/DAD2 receptor recognizes and hydrolyses different SL compounds, depends on the stereospecificity of SLs compounds (reviewed by Flematti et al., 2016) which thus play a crucial role in SLs perception and plant responses.

Although *AtD14* expression is found in all major plant organs, it still shows a high tissue specificity. For example in roots, expression of *AtD14* was mainly in the vascular cylinder of the differentiation and elongation zones, whereas in leaves or cotyledons, a higher expression of *AtD14* was observed in the phloem (Chevalier et al., 2014). Intriguingly, the pattern of *AtD14* gene expression does not correspond to the AtD14 protein presence. For example, the AtD14 protein was found in nuclei of root meristem and rhizodermal cells, which were without relevant gene expression, indicating that either the mRNA or the D14/DAD2 protein is transported between the cells. Indeed, grafting studies confirmed that the D14/DAD2 protein is able to move between cells by short distance transport (Hamiaux et al., 2012; Chevalier et al., 2014).

Abundance of *AtD14* mRNA did not change after treatment with auxin or the synthetic SL analog GR24, as well as during axillary bud development (Chevalier et al., 2014). It was therefore postulated that regulation of receptor abundance occurs at the protein level. Indeed, treatment of *A. thaliana* seedlings with GR24 resulted in a decreased AtD14 protein content (Chevalier et al., 2014). X-ray crystallography and hydrogen-deuterium exchange mass spectrometry (HDX) of the rice protein OsD14 and its conformational change after binding to GR24 molecules showed that binding to GR24 destabilizes the OsD14 (Zhao et al., 2015). This was the first indication of a phytohormone degrading its own receptor and affecting its own perception. It would be worth to investigate if this unexpected relation between signal molecule and receptor is indeed specific for SLs or whether it presents a more general mode of action among phytohormones.

## SL Signal Conversion

Degradation of targeted proteins via the ubiquitin-proteasome pathway plays a crucial role in the signaling pathway of most phytohormones (Wang C. et al., 2015). The central element of this system is the SKP1-CULLIN-F-BOX complex (SCF). SL perception involves recognition and binding of target proteins by F-Box proteins which are subsequently bound by Skp1, before Cullin, the main structural component of the SCF complex, connects the complex to ubiquitin ligase (Larrieu and Vernoux, 2015). Since the F-box protein component renders specificity to the whole CSF complex, each hormone/signaling molecule may have its own exclusive F-box protein component. The protein recognized by the F-box protein is ubiquitinated thus marking it for proteasomal degradation.

In studies on the *A. thaliana* mutant *max2* and the rice mutant *d3* an F-box protein involved in SL signaling was identified that was also part of an SCF ubiquitin ligase protein complex (Stirnberg et al., 2002; Ishikawa et al., 2005). In *A. thaliana* MAX2 forms the SCF complex together with AtCullin1 and ARABIDOPSIS SERINE/THREONINE KINASE 1 (ASK1), whereas in rice the D3 protein interacts with OsCullin1 and ORYZA SATIVA SKP1-LIKE1/5/20 (OSK1/5/20) (Stirnberg et al., 2007; Zhao et al., 2014) (**Figure 2**). Similar to other components of the SLs signaling pathway, MAX2/D3 has a nuclear localization and the expression patterns of genes encoding this protein were similar to those observed for *D14/DAD2* (Stirnberg et al., 2007; Zhao et al., 2014). The interaction between MAX2/D3 and D14/DAD2 was experimentally confirmed, and was shown to be promoted by the presence of SLs (Hamiaux et al., 2012; Zhao et al., 2014). Bimolecular fluorescence complementation analysis in rice protoplasts confirmed a GR24-mediated interaction between D3 and D14 within the nucleus (Zhao et al., 2014). The properties of this interaction which is mediated by SLs and depends on the SL concentration, is also affected by the SL stereoisomers involved (Zhao et al., 2015).

While certain components of the SL signaling pathway appear specific for SLs, the MAX2/D3 element is also involved in karrikin signal transduction. It is suggested that MAX2 may be part of different SCF complexes that are able to bind a range of substrates/repressors (Nelson et al., 2011). Observations in rice, where D3 interacts with at least three different OSKs, confirm the hypothesis that MAX2 can interact with multiple SCF complexes (Zhao et al., 2014). Moreover, it has been shown that MAX2 is also involved in the degradation of BRASSINAZOLE-RESISTANT1 (BES1), the transcriptional effector of the phytohormone class of brassinosteroids (Wang et al., 2013).

A phylogenetic analysis revealed similarity of MAX2/D3 to the auxin receptor TRANSPORT INHIBITOR RESPONSE1 (TIR1) (Dharmasiri et al., 2005) and the jasmonate receptor CORONATINE INSENSITIVE1 (COI1) (Sheard et al., 2010). Although there is no evidence that MAX2/D3 acts as a SL receptor, it cannot be excluded that this protein may recognize other signaling molecules, such as karrikins, since the *A. thaliana max2* mutant showed a karrikin-resistant phenotype (Nelson et al., 2011).

All these data indicate that MAX2/D3 is probably involved in multiple signaling pathways and/or is a connector between SL perception and other phytohormones. This is a reason why to investigate the role of SLs in different aspects of plant growth and development it is better to use the SL-synthesis mutants or mutants in *D14/DAD2* genes, that encoding receptor specific only for SLs. Whereas the results obtained for *max2/d3* mutants might be related to their multiple role in plant signaling network. Now the identification of specific molecules recognized by MAX2/D3, as well as the identification of targets for the SCF^MAX2/D3^ complex is necessary to uncover the comprehensive role of this protein in the plant signaling network.

## SL Signaling

The first SL repressor identified was D53 from rice (Zhou et al., 2013). Similar to other components of the SL signaling pathway D53 was discovered in a screening of SL-insensitive mutants displaying semi-dwarf phenotypes and higher number of tillers compared to their wild-type counterparts. Interestingly both, *d53* mutants and wild-type plants overexpressing *OsD53* showed increased branching, suggesting that the mutation in *D53*, i.e., a deletion of five amino acids, confers gain-of-function. The role of D53 in repressing the SL signal was confirmed by the lower number of tillers in *d53* plants with reduced expression of *D53* (Zhou et al., 2013). Recently, three orthologous of D53 identified in *A. thaliana* were also found to act as suppressors in SL signaling and named SUPRESSOR OF MAX2-LIKE6 to 8 (SMXL6 to 8) (Soundappan et al., 2015; Wang L. et al., 2015). First report indicated that all three genes function redundantly as shown by the fact that a reduced branching phenotype was only observed in the triple mutant *smxl6/7/8* (Wang L. et al., 2015). However, recently it was shown that the presence of a stabilized form of SMXL7 under native promotor, resulted in a phenotype characteristic for SL mutants (Liang et al., 2016). Thus the question if all three repressors function redundantly remains still open.

The gene products of *D53*, *SMXL6* to *SMXL8* are localized in the nucleus. The presence of SL molecules was found to promote the interaction between these proteins and the receptor D14 (Zhou et al., 2013; Wang L. et al., 2015). At the same time SLs also induce fast proteasome-mediated degradation of D53 (Zhou et al., 2013), SMXL6 (Wang L. et al., 2015), and SMXL7 (Soundappan et al., 2015). Since degradation of D53 was not observed in *d3*, *d14* and *d53* mutants, it was concluded that the presence of the D3-D14-D53 complex is necessary for the degradation of SL repressors (**Figure 2**). Although interactions between D14/AtD14, D3/MAX2, and D53/SMXL6 to 8 have been confirmed, the interaction between SMXL6 and MAX2 does not require the presence of D14 and the interaction between SMXL6 and AtD14 does not require MAX2 (Wang L. et al., 2015).

The SL repressors found in rice and *A. thaliana* contain a highly conserved ethylene-responsive element binding factor-associated amphiphilic repression (EAR) motif of five amino acids (F/L-D-L-N-L). This motif has been postulated to interact with the transcriptional corepressors TOPLESS and TOPLESS-RELATED PROTEINS (TPR2) (Zhou et al., 2013; Ke et al., 2015; Soundappan et al., 2015) (**Figure 2**). Using a yeast-two hybrid and Co-Immunoprecipitation assays, Wang L. et al. (2015) were able to confirm the interaction between SMXL6 to 8 and TPR2 *in vivo*. In *A. thaliana* it was recently shown that SMXL7, D14, and MAX2 interact in the nucleus in an SL-dependent manner (Liang et al., 2016).

Presence of at least three SL-repressors in *A. thaliana* indicates a diverse regulation of the SLs signaling pathway and thus increasing the range of influences on different aspects of plant development. Studies on individual SMXLs and identification of genes regulated by SCF complexes containing different repressors, will confirm this hypothesis.

## SL-Elicited Responses

The final confirmation that D53/SMXL6 to 8 act as SL repressors was provided by gene expression analysis. Until now only one class of transcription factors (TFs), the TEOSINTE BRANCHED1/CYCLOIDEA/PROLIFERATING CELL FACTO-R1 family (TCP), has been described as downstream component in SL signaling (Braun et al., 2012) (**Figure 2**). Representatives of TCP TFs have been found in rice (FC1, FINE CULM1) and *A. thaliana* (AtBRC1, BRANCHED1), and their expression has been observed in axillary buds. Both *AtBRC1* and *FC1* were upregulated after treatment with GR24, confirming their role in SL-mediated plant responses (Aguilar-Martínez et al., 2007; Minakuchi et al., 2010). Expression of *AtBRC1* was down-regulated in SL-biosynthesis mutant *max3* and SL-signaling mutant *max2* but up-regulated in triple mutant *smxl6/7/8* (Soundappan et al., 2015; Wang L. et al., 2015). Similar results were found for *HB53*, one of the known target genes of *AtBRC1*, which was elevated in *smxl6/7/8* plants (Wang L. et al., 2015).

Knowledge on the interactions of SLs repressors and corepressors will allow to predict which TFs might be regulated by SLs, thus enabling a forecast to the plant response to SLs on the transcriptional level. Moreover, the comparative transcriptome analysis of individual *smxl* mutants might also reveal if all repressors function redundantly or not.

## Common and Unique Features of SL Perception

Based on the genetic analysis of SL-insensitive mutants in rice and *A. thaliana*, three main players in SL signal transduction have already been identified: receptor D14/DAD2, repressor D53/SMXL6 to 8 and F-box protein MAX2/D3, which is a part of the SCF complex. The SL signaling pathway shares similarities with those of other phytohormones. D14/DAD2, the receptor of SLs resembles the gibberellin receptor GID1 (Griffiths et al., 2006; Arite et al., 2009; Hamiaux et al., 2012). Furthermore, proteasome-mediated degradation of the repressor by the SCF complex is a well-known mechanism of phytohormone-regulated gene expression (Wang L. et al., 2015).

Other aspects of the SLs signaling pathway seem very specific though. In contrast to the closely related gibberellin receptor GID1, the SL receptor D14/DAD2 is able to hydrolyse its receptor molecules (Hamiaux et al., 2012). Even more intriguing is that during the hydrolysis of SLs the conformation of D14 also changes which initiates the destabilization of this protein (Chevalier et al., 2014). Together with the degradation of D53/SMXL6 to 8 the perception of SLs constitutes a unique phenomenon among plant hormones involving the successive degradation of signal molecule, receptor, and downstream effector.

## Author Contributions

The author confirms being the sole contributor of this work and approved it for publication.

## Conflict of Interest Statement

The author declares that the research was conducted in the absence of any commercial or financial relationships that could be construed as a potential conflict of interest.
